# Supplementary material for: Auditory information enhances post-sensory visual evidence during rapid multisensory decision-making
Source: Nat Commun. 2020 Oct 28;11:5440. doi: 10.1038/s41467-020-19306-7 (PMC7595090; doi:10.1038/s41467-020-19306-7)
Supplement: Supplementary file 2 — Reporting Summary [file 41467_2020_19306_MOESM2_ESM.pdf]

nature research

Corresponding author(s): Marios Philiastides, Leon Franzen

Last updated by author(s): Sep 20, 2020

Reporting Summary

Nature Research wishes to improve the reproducibility of the work that we publish. This form provides structure for consistency and transparency in reporting. For further information on Nature Research policies, seeAuthors & Referees and theEditorial Policy Checklist .

Statistics

For all statistical analyses, confirm that the following items are present in the figure legend, table legend, main text, or Methods section.

n/a	Confirmed	
☐	☒	The exact sample size (n) for each experimental group/condition, given as a discrete number and unit of measurement
☐	☒	A statement on whether measurements were taken from distinct samples or whether the same sample was measured repeatedly
☐	☒	The statistical test(s) used AND whether they are one- or two-sided *Only common tests should be described solely by name; describe more complex techniques in the Methods section.*
☐	☒	A description of all covariates tested
☐	☒	A description of any assumptions or corrections, such as tests of normality and adjustment for multiple comparisons
☐	☒	A full description of the statistical parameters including central tendency (e.g. means) or other basic estimates (e.g. regression coefficient) AND variation (e.g. standard deviation) or associated estimates of uncertainty (e.g. confidence intervals)
☐	☒	For null hypothesis testing, the test statistic (e.g. F, t, r) with confidence intervals, effect sizes, degrees of freedom and P value noted *Give P values as exact values whenever suitable.*
☐	☒	For Bayesian analysis, information on the choice of priors and Markov chain Monte Carlo settings
☐	☒	For hierarchical and complex designs, identification of the appropriate level for tests and full reporting of outcomes
☐	☒	Estimates of effect sizes (e.g. Cohen's d, Pearson's r), indicating how they were calculated

Our web collection on statistics for biologists contains articles on many of the points above.

Software and code

Policy information about availability of computer code

Data collection	Brain Vision Recorder (BVR; version 1.10, Brain Products GmbH, Germany); PsychoPy presentation software (version 1.83.04); The stimuli associated with ref. The scrambled face and car stimuli used in this study can be downloaded from: http://mphiliastides.org/files/download/228. The original (unscrambled) face stimuli can be obtained from: https://faces.kyb.tuebingen.mpg.de/index.php [ref 88].
Data analysis	MATLAB (version 2015b, The MathWorks, 2015, Natick, Massachusetts); RStudio (version 1.2.1335); Analysis code was build on code obtained from the Figshare and GitHub repositories: [https://figshare.com/articles/Modern_graphical_methods_to_compare_two_groups_of_observations/4055970], [https://github.com/GRousselet], and [https://figshare.com/articles/A_few_simple_steps_to_improve_the_description_of_group_results_in_neuroscience/3806487]. Linear Discriminant Analysis (LDA) code can be found here: [http://liinc.bme.columbia.edu/wp-content/uploads/lr1.2_plugin.tar.gz?x99316].

For manuscripts utilizing custom algorithms or software that are central to the research but not yet described in published literature, software must be made available to editors/reviewers. We strongly encourage code deposition in a community repository (e.g. GitHub). See the Nature Research guidelines for submitting code & software for further information.

Data

Policy information about availability of data

All manuscripts must include a data availability statement. This statement should provide the following information, where applicable:

- Accession codes, unique identifiers, or web links for publicly available datasets
- A list of figures that have associated raw data
- A description of any restrictions on data availability

Source data are provided with this paper. The full neural and behavioural data required to reproduce the main analyses supporting this work are available from the study's Open Science Framework repository [https://osf.io/rhx6y/]. The raw EEG dataset is available from the corresponding authors upon request.

Field-specific reporting

Please select the one below that is the best fit for your research. If you are not sure, read the appropriate sections before making your selection.

☒ Life sciences ☐ Behavioural & social sciences ☐ Ecological, evolutionary & environmental sciences

For a reference copy of the document with all sections, see nature.com/documents/nr-reporting-summary-flat.pdf

Life sciences study design

All studies must disclose on these points even when the disclosure is negative.

Sample size	The following can also be found in the Methods section under "Participants". We estimated a minimum sample size of 35 participants by an a priori power analysis for a fixed linear multiple regression model with two predictors, a medium effect size of 0.5, an alpha of .05, and a power of .95. In the case of the present study, the two predictors were modality (i.e., visual and audiovisual) and visual coherence level of the employed images. To fulfill the estimated sample size requirements, we tested 40 participants (male = 18, female = 22; Mean age = 23.85, SD = 5.47).
Data exclusions	All exclusion criteria were established before any analyses were performed. Participant inclusion and exclusion criteria are described in the Methods section. Trial exclusion criteria (to eliminate lost trials or trials exceeding pre-established response deadlines) are also stated. For example, we excluded trials where participants exceeded the response time limit of 1.5 seconds, indicated a response within less than 300ms after onset of the stimulus or the EEG signal exceeded a maximum amplitude of 150 μV during the trial as data with these properties would not constitute behavioural data of interest for rapid decision-making, unrealistic or confounded by substantial neural noise. Excluded trials make up less than 1% of trials. These are fully explained within the corresponding parts of the Methods section.
Replication	EEG analysis pipeline was performed on individual participants using trial-based cross validation such that each participant became their own replication unit.
Randomization	Randomization of groups/participants is not applicable due to a fully crossed experimental design. Order of trials and the order of the first two training tasks was randomized.
Blinding	Not applicable as we only employed one group of participants who took part in all conditions.

Reporting for specific materials, systems and methods

We require information from authors about some types of materials, experimental systems and methods used in many studies. Here, indicate whether each material, system or method listed is relevant to your study. If you are not sure if a list item applies to your research, read the appropriate section before selecting a response.

Materials & experimental systems

n/a	Involved in the study	
☒	☐	Antibodies
☒	☐	Eukaryotic cell lines
☒	☐	Palaeontology
☒	☐	Animals and other organisms
☐	☒	Human research participants
☒	☐	Clinical data

Methods

n/a	Involved in the study	
☒	☐	ChIP-seq
☒	☐	Flow cytometry
☒	☐	MRI-based neuroimaging

Human research participants

Policy information about studies involving human research participants

Population characteristics	We sampled 40 participants living in the United Kingdom at time of participation (male = 18, female = 22; Mean age = 23.85, SD = 5.47). All participants were right-handed with normal or corrected-to-normal vision and no self-reported history of neurological disorders. This information is provided to the reader in the Methods section under Participants.
Recruitment	Participants were recruited using standard procedures from a large recruitment pool with more than 5000 registered individuals living in the United Kingdom capturing a large spectrum of gender, ethnicity, and race. Participation was entirely voluntary. No aging effects are likely in our sample consisting of young adults.
Ethics oversight	This study was approved by the ethics committee of the College of Science and Engineering at the University of Glasgow (CSE 300150102) as stated in the first paragraph of the Methods section.

Note that full information on the approval of the study protocol must also be provided in the manuscript.
